# Supplementary material for: Evidence for causal top-down frontal contributions to predictive processes in speech perception
Source: Nat Commun. 2017 Dec 18;8:2154. doi: 10.1038/s41467-017-01958-7 (PMC5735133; doi:10.1038/s41467-017-01958-7)
Supplement: Supplementary file 1 — Supplementary Information [file 41467_2017_1958_MOESM1_ESM.pdf]

Supplementary Figures

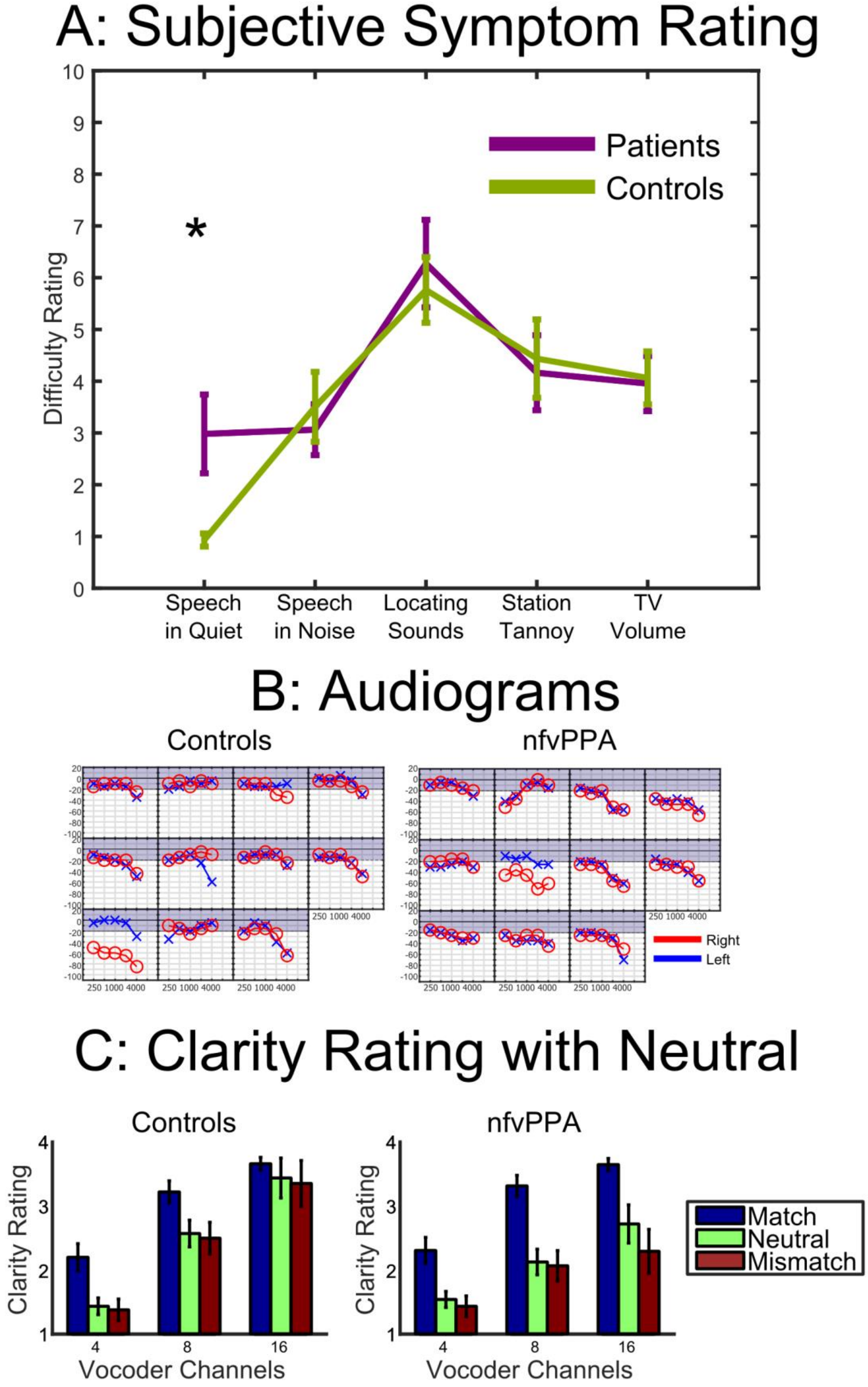

Supplementary Figure 1: A: Self-rated listening difficulty for patients and controls for four different listening scenarios, and the related question: “how loud do people tell you your TV is?” B: Pure tone audiograms for each individual participant. C: Group averaged clarity ratings from the repetition of experiment 1 (figure 1A) with the addition of a neutral written cue (a row of XXXX).

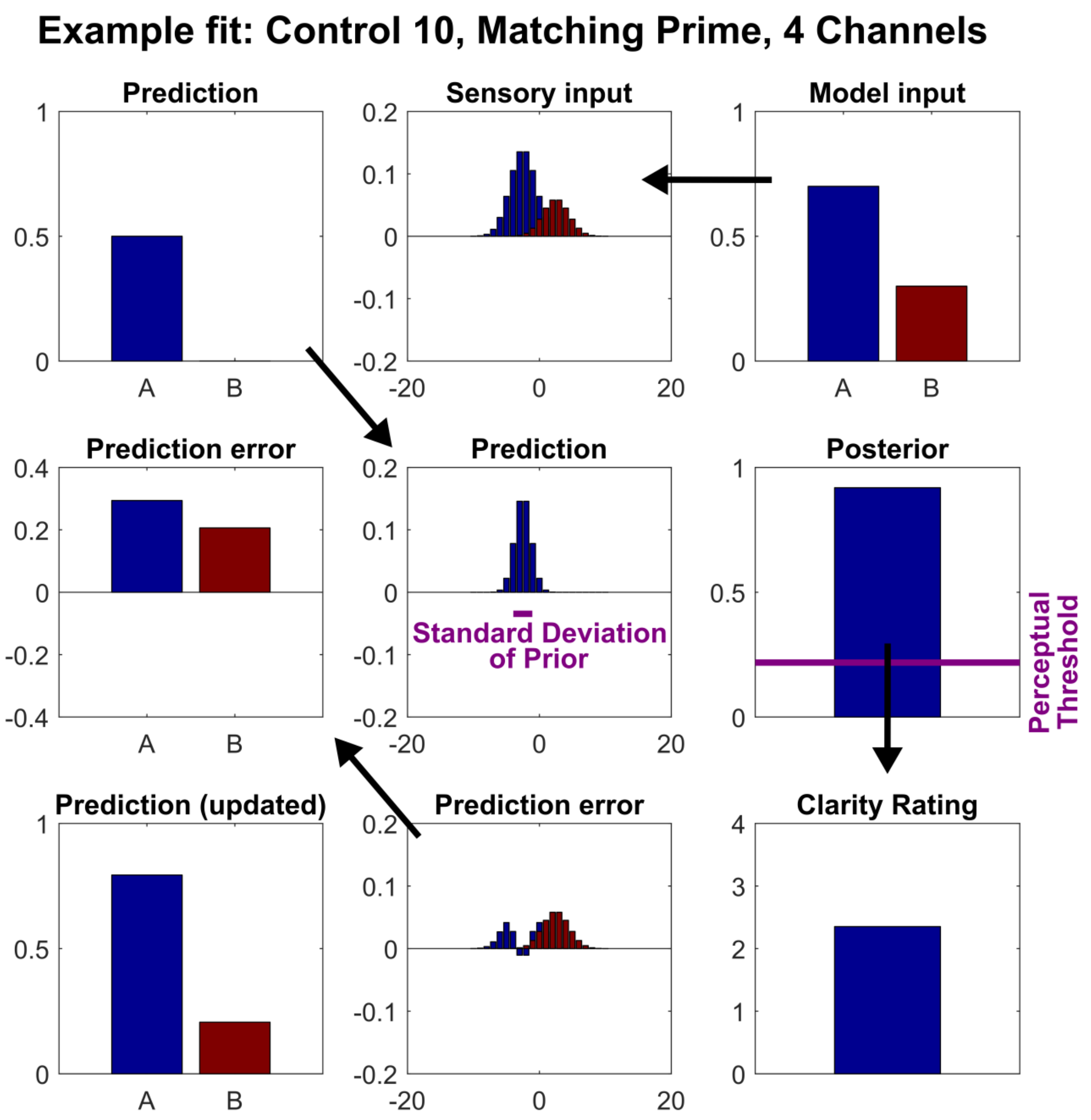

Supplementary Figure 2: Example Bayesian model fits for a single subject in a single condition (in this case control 10 for 4 channel vocoded speech and matching written cue and vocoded word) after [2]. The standard deviation of the prior and the perceptual threshold were individually optimised to best model the clarity ratings from experiment 1. Upper left: the prediction, in this case a 50% chance of the cued word (A) and an approximately zero chance of any other given word (B). Middle left: the total area under the curve of the prediction error fed back to frontal regions from temporal regions. Bottom left: the updated prediction, from which an A vs B discrimination might be performed. Upper centre: the modelled activity in a set of 21 neural units representing phonological feature categories; a weighted function of the upper right panel. Middle centre: the modelled prediction for activity in the set of 21 neural units representing phonological feature categories; a weighted function of the upper left panel. Bottom centre: The prediction error in each of the 21 modelled neural units, calculated by subtracting the prediction from the sensory input. Upper right: the probability density for the sensory identification of the cued word (A) vs any other word (B); this was defined for each subject individually as the percentage above chance in experiment 2 at the number of vocoder channels being modelled. Middle right: the area under the curve of the posterior distribution, calculated as the sum of the area under the curve of the sensory input and a weighted

product of the sensory input and prior precision, with the weighting determined by the congruency of the written cue. Bottom right: the modelled clarity rating, calculated as the height of the posterior above perceptual threshold, individually normalised to a 1-4 rating scale.

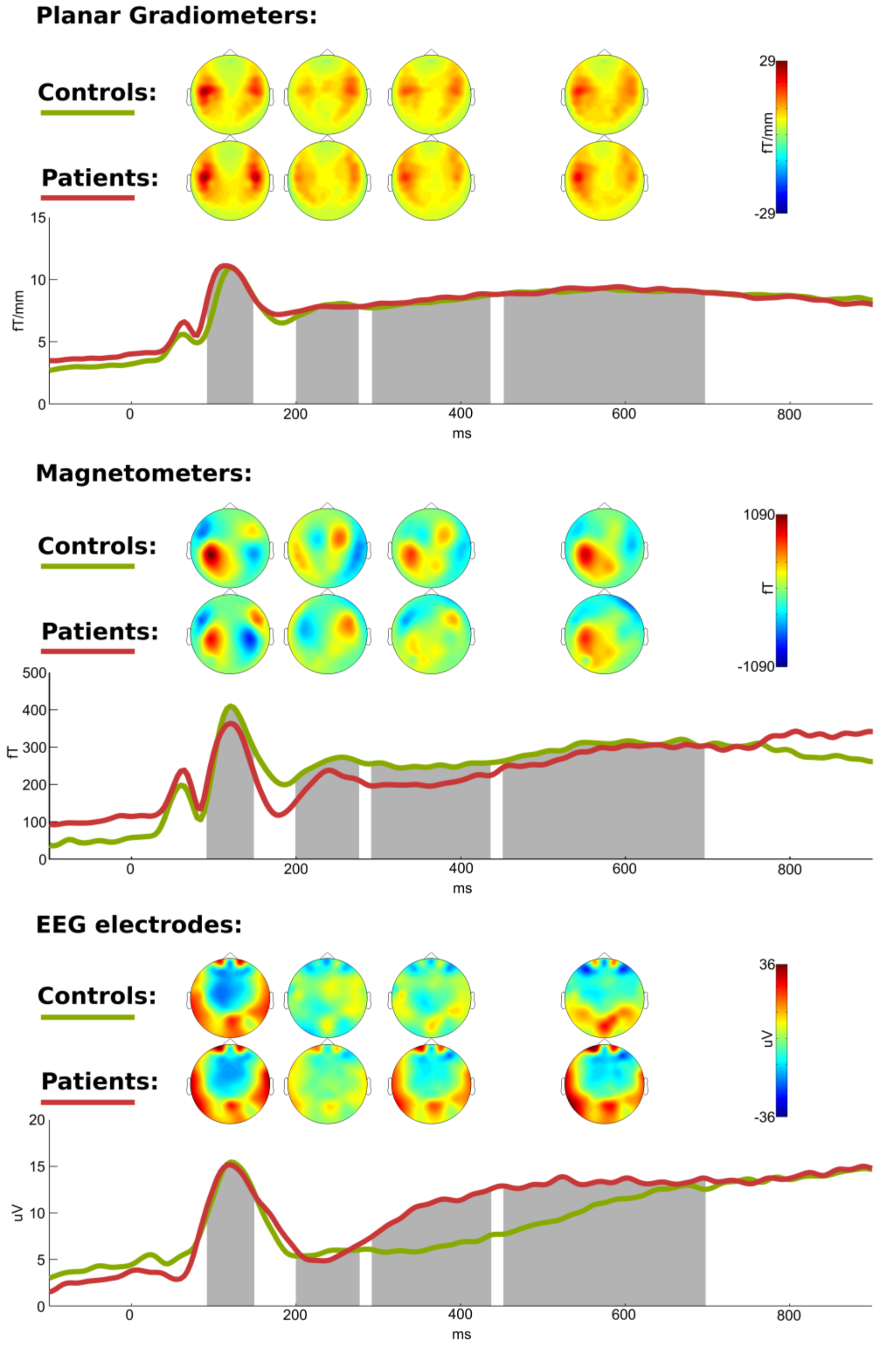

Supplementary Figure 3: Root mean square averaged evoked response across all sensors for each modality, after averaging across conditions and participants. The time windows over which the evoked brain sources were reconstructed are depicted by the areas shaded in gray (90-150ms, 200-280ms, 290-440ms, 450-700ms). Scalp topographic plots display the average evoked response within each window.

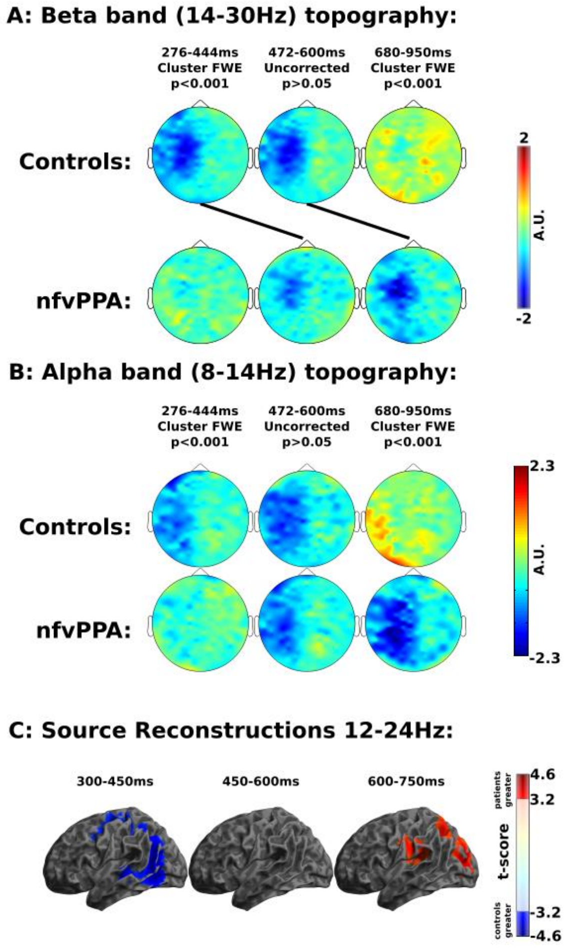

Supplementary Figure 4: A: Scalp topologies for the group by congruency interaction in the beta frequency band. B: Scalp topologies for the group by congruency interaction in the alpha frequency band. C: eLORETA source reconstructions for 300-450ms, 450-600ms and 600-750ms, corresponding to time windows displaying a greater effect of congruency in controls, no group by congruency interaction, and a greater effect of congruency in nfvPPA. Only left hemispheres are shown, as no significant right sided sources were demonstrated.

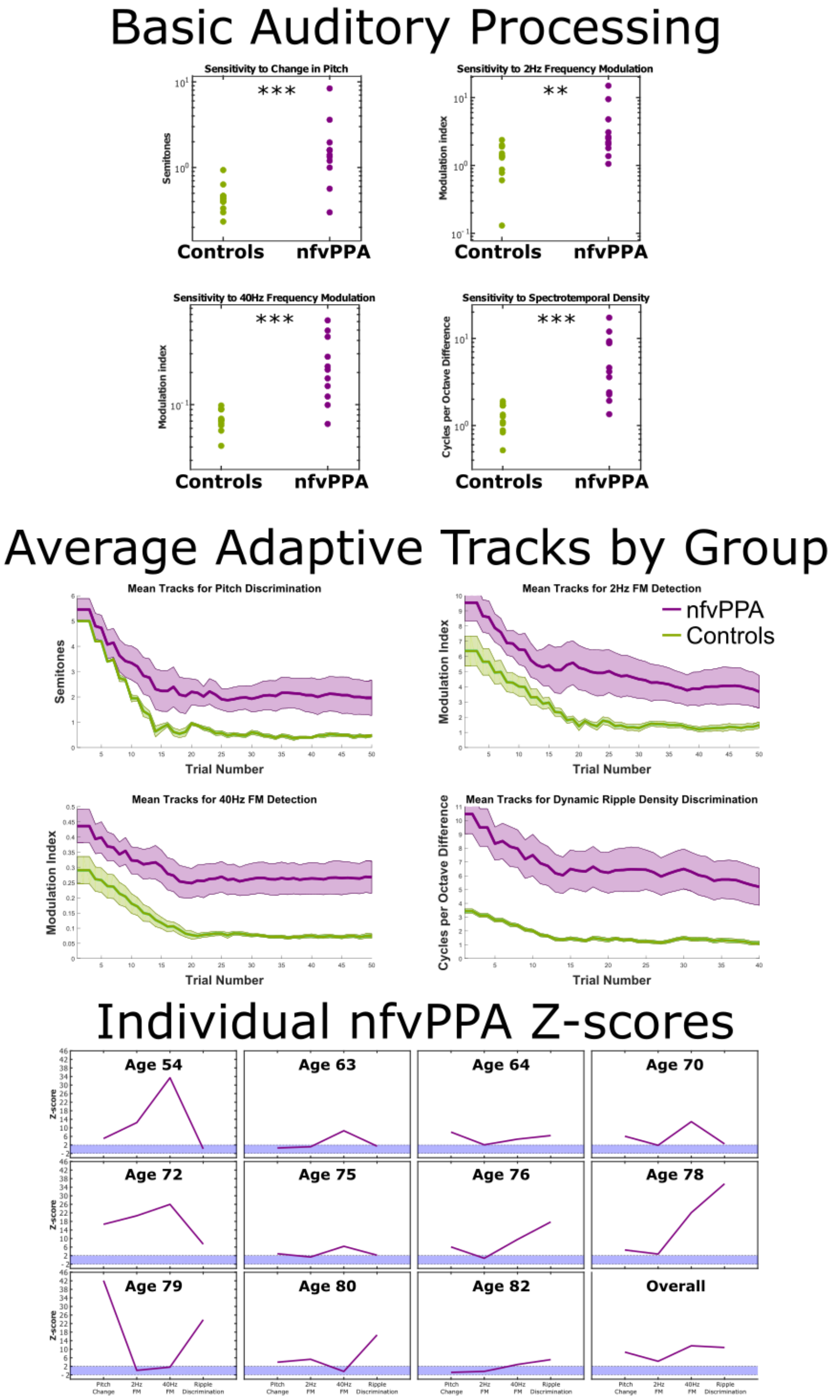

Supplementary Figure 5: Basic auditory processing, after Grube et al. [22]. The tasks employed were pitch change detection, 2Hz FM detection, 40Hz FM detection, and dynamic ripple density discrimination.

Supplementary Tables

Supplementary Table 1: Voxel-based morphometry regions of significant atrophy in nfvPPA, corresponding to figure 1E. Output of an SPM thresholded at peak FWE p<0.05. To avoid an over-long table, only clusters of >= 500 voxels are listed here.

cluster	cluster	peak	peak	MNI Co-ordinates			
p (FWE)	voxels	p (FWE)	t-score	x	y	z	Neuromorphometrics region
0.001	751	0	6.95	-14	25	53	Left Superior Frontal Gyrus
0	1675	0	6.92	11	8	58	Right Supplementary Motor Cortex
		0.001	6.52	5	-1	49	Right Supplementary Motor Cortex
		0.001	6.5	4	0	62	Right Supplementary Motor Cortex
0.001	879	0.001	6.65	-35	17	7	Left Frontal Operculum
		0.021	5.46	-49	19	12	Left Opercular Part of Inferior Frontal Gyrus
0.002	638	0.001	6.49	31	-4	56	Right Precentral Gyrus
		0.018	5.53	28	2	49	Right Middle Frontal Gyrus
		0.018	5.52	19	0	58	Right Superior Frontal Gyrus
0	1069	0.002	6.27	40	5	9	Right Central Operculum
		0.004	6.05	37	20	6	Right Frontal Operculum

Supplementary Table 2: Repeated measures ANOVA of evoked source power in frontal and temporal voxels of interest over the whole post auditory epoch. Bold, red rows indicate statistically significant results.

	DF	F	p
Group	1,134	1.1	0.317
**Source**	**1,134**	**2003.1**	**<0.001**
**Condition**	**1,134**	**5.1**	**0.037**
Vocoder Channels	2,134	0.0	0.970
Individual (Group)	18,134	1.1	0.383
Group*Source	1,134	0.1	0.816
Group*Condition	1,134	0.4	0.550
Group*Vocoder Channels	2,134	0.8	0.433
**Source*Condition**	**1,134**	**60.1**	**<0.001**
Source*Vocoder Channels	2,134	0.1	0.884
Condition*Vocoder Channels	2,134	1.8	0.165
**Group*Source*Condition**	**1,134**	**11.8**	**0.001**
**Source* Individual (Group)**	**18,134**	**76.6**	**<0.001**
**Condition * Individual (Group)**	**18,134**	**4.9**	**<0.001**
Vocoder Channels * Individual (Group)	36,134	1.2	0.227

Supplementary Results

Basic Auditory Processing

In this larger cohort we replicated the finding of Grube, et al. [1], namely that patients with nfvPPA perform very poorly at some tasks of basic auditory processing. This does not seem to have a trivial explanation like an inability to sustain attention or yes/no confusion, as the individual adaptive tracks have a similar shape in patients and controls (supplementary figure 5), with consistent correct responses in 'easy' trials and, once threshold is reached, flat profiles maintained for the remainder of a run. The pattern of performance was highly variable between individuals, but highly consistent within individuals; as can be seen in the individual Z-scores for each task, some patients were able to consistently perform some discrimination tasks in the normal range, while being dozens of standard deviations poorer than the mean in other tasks. Patients who performed well on a particular task continued to perform well if it was repeated but, even after repeated practice, they remained unable to perform well on tasks that they had previously found difficult.

Supplementary Discussion

Basic Auditory Processing in nfvPPA

In our larger cohort, we confirmed the previous suggestion that deficits in auditory processing are over-represented in patients with nfvPPA (supplementary figure 5) [1,2]. On the face of it, this seems a surprising finding as the Bayesian VBM provides evidence for no atrophy in primary auditory regions (figure 1E), and at post mortem patients with nfvPPA do not display disproportionate pathology in either primary auditory cortex or auditory brainstem nuclei. Further, it is known that patients with progressive supranuclear palsy, who do have severe brain stem atrophy, continue to display complex auditory psychophysical effects late in disease [3]. Similarly, our patients with nfvPPA demonstrated good performance at identifying vocoded words, performing almost as well as controls (figure 2D). Finally, the pattern of psychophysical deficits was observed to be highly variable (supplementary figure 5); impairment of a bottom-up perceptual process would predict a consistent profile of performance that might vary in severity, while what we observe is that all individuals perform very poorly on some tasks, but the relative difficulty of the tasks varies between individuals. A higher level, cortical explanation must therefore be invoked to account for these psychophysical findings. This effect has previously been understood in terms of impaired working memory [1], but it is worth considering whether it might also be explained by the abnormalities of predictive coding demonstrated here.

Basic auditory processing is traditionally assessed with two- or three-alternative forced choice paradigms, with the difference between exemplars adaptively modified to track a given performance percentile [4]. A predictive coding model can also be applied to this experimental context, in which subjects make a decision based on the location of the peak of their posterior in the perceptual dimension of interest. The distribution of this posterior is based on a prediction that is modified by prediction error induced by sensory input. For example, in a task where one is asked to detect the presence of a pitch change the subject might listen to the first pitch and then set up a prediction that the second pitch would be unchanged. A decision would be made by performing two-point discrimination on the peak locations of prior and posterior distributions. If all subjects establish similar predictions, the accuracy of this decision process is dependent only on the precision of the sensory input. If, however, subjects with nfvPPA make more precise predictions, the perceptual distance between prior and posterior would be reduced, leading to poor discriminatory performance even though the sensory input is unchanged. This also explains the lack of a hierarchical relationship in performance profiles (supplementary figure 5); for example to discriminate the density of dynamic ripples it is necessary to be able to process frequency modulations, and yet some patients were able to perform within the normal range at discriminating ripples whilst seemingly unable to detect its building blocks (i.e. frequency modulation).

This explanation in terms of abnormally precise predictions is not exclusive of that in terms of impaired working memory (which could be modelled here as a drift in the location of the prior distribution over time), and indeed both processes could be occurring simultaneously. The argument we make is simply that it is possible that impaired predictive coding, of itself, is a sufficient explanation for measured impairments in basic sound discrimination in nfvPPA.

Differences in implicit learning

To address the question of whether our findings can be accounted for by differences between groups in implicit learning, we exploited the fact that the repetition of experiment 1, with the addition of neutral primes, was always performed after the MEG session and after experiment 2. While mismatch clarity ratings in nfvPPA for 8 and 16 channel speech were very slightly higher at this repetition than during the MEG session, which we speculate may reflect a combination of implicit learning [5] and a decrease in the likelihood of prior congruency from 50% to 33%, the large group by congruency interaction remained. This implies that perceptual learning of degraded speech across the experiment cannot account for the difference in patients between low mismatch and neutral clarity ratings in experiment 1 and good vocoded word report in experiment 2.

Supplementary References

1 Grube, M. *et al.* Core auditory processing deficits in primary progressive aphasia. *Brain*, doi:10.1093/brain/aww067 (2016).

2 Goll, J. C. *et al.* Non-verbal sound processing in the primary progressive aphasias. *Brain* **133**, 272-285, doi:Doi 10.1093/Brain/Awp235 (2010).

3 Hughes, L. E. *et al.* The binaural masking level difference: cortical correlates persist despite severe brain stem atrophy in progressive supranuclear palsy. *J. Neurophysiol.*, jn 00062 02014, doi:10.1152/jn.00062.2014 (2014).

4 Levitt, H. Transformed up-down methods in psychoacoustics. *J. Acoust. Soc. Am.* **49**, Suppl 2:467+ (1971).

5 Sohoglu, E. & Davis, M. H. Perceptual learning of degraded speech by minimizing prediction error. *Proceedings of the National Academy of Sciences* **113**, E1747-E1756 (2016).
